# Oculogyric Crisis Following Dosage Increase of Aripiprazole: A Case Report

**DOI:** 10.7759/cureus.65295

**Published:** 2024-07-24

**Authors:** Nikhale Malik, Sally Othman, Apoorv Tiwari, Sushmita Mantravadi, Syeda Salari, Abed Dabaja, Sujata Kambhatla

**Affiliations:** 1 Internal Medicine, Garden City Hospital, Garden City, USA

**Keywords:** extrapyramidal side effects, atypical antipsychotic, rhabdomyolysis, acute dystonic reaction, aripiprazole

## Abstract

Oculogyric crisis (OGC) is a form of acute dystonia with involuntary conjugated ocular gaze in a fixed position. Common causes include metabolic disorders and medications, primarily typical antipsychotics. A 24-year-old male with a history of persistent depressive disorder and possible bipolar disorder was admitted to the psychiatric unit due to suicidal ideation. He was started on aripiprazole 5 mg and subsequently increased to 10 mg due to uncontrolled mood disorder. The patient subsequently developed OGC, which resolved with benztropine and diphenhydramine. This case report highlights aripiprazole-induced OGC and prompt management with benztropine and first-generation antihistamines.

## Introduction

Oculogyric crisis (OGC) is a form of acute dystonia in which the patient involuntarily experiences conjugate gaze in a fixed position, typically upwards [1]. These episodes can last from seconds to hours. Etiologies of OGC include medication-induced (primarily dopamine-blocking agents), focal neurologic lesions, post-encephalitic parkinsonism, and certain metabolic and genetic disorders including Wilson’s disease, neurosyphilis, and multiple sclerosis [2-4].

While the exact pathophysiology is unclear, there is a clear association with dopaminergic hypoactivity and the development of OGC [5]. Consequently, it is also commonly seen in patients with lesions in the nigrostriatal pathway as this would disrupt dopamine synthesis. Common medications associated with OGC include high-potency neuroleptic agents such as haloperidol, trifluoperazine, and fluphenazine.

Aripiprazole is a third-generation atypical antipsychotic that is commonly used to treat psychosis in patients with schizophrenia and bipolar disorder; however, more recently, it is being used to treat refractory depression [6]. At the molecular level, it acts as a partial agonist at the 5HT-1a and D2 receptors and an antagonist at the 5HT-2a receptor [7]. However, in areas of high dopamine, such as the mesolimbic pathway, aripiprazole will act as a functional dopamine antagonist [8].

A review of the available literature shows that 1.8% of all OGC cases are induced by second-generation antipsychotics [5]. However, given its rarity, there is scant data in the literature quantifying the percentage of OGC induced by third-generation antipsychotics. In this article, we discuss the case of a 24-year-old male who developed acute OGC following an increase in the dosage of aripiprazole.

## Case presentation

A 24-year-old male with a history of alcohol abuse and mood disorder was brought into the emergency department due to suicidal intent. Vital signs on admission were a temperature of 98.2°F, blood pressure of 120/82 mmHg, heart rate of 97 per minute, respiratory rate of 16 per minute, and oxygen saturation of 97% on room air. He admitted to being depressed and under extreme stress. He had suicidal ideations, but no definite plan for self-harm. He was following up with counseling at an outpatient behavioral health facility. He was not on any psychiatric medications.

On examination, the patient was alert and oriented to time, place, and person. His cranial nerve examination was unremarkable. He was cooperative. His mood was anxious, labile, and impulsive. The patient was not acutely psychotic. The psychiatrist diagnosed the patient with persistent depressive disorder with suicidal thoughts and possible bipolar disorder. The patient was admitted to the inpatient psychiatric unit given his labile mood and suicidal ideations.

The patient was placed on suicide precautions in the psychiatric unit. He was started on aripiprazole 5 mg daily, benztropine 1 mg daily, and duloxetine 20 mg twice daily. There was no improvement in the patient’s mood or behavior after four days on this medication regimen. Aripiprazole was then increased from 5 mg to 10 mg daily.

The following day, the rapid response team was activated as the patient was found face down in the bathroom by nursing staff. The circumstances surrounding him being face down in the bathroom were unclear, and he had no visible evidence of a traumatic fall. Vital signs at that time showed blood pressure of 130/70 mmHg, heart rate of 110 beats per minute, respiratory rate of 18, and oxygen saturation of 99% on room air. The patient was no longer verbally communicating. He had a sustained right-sided conjugate upward gaze. No other dystonic reactions were appreciated. During the episode, the patient was responding to commands as he was weakly squeezing the examiner’s fingers when requested. He was noted to have urinary incontinence. A fingerstick glucose was within normal limits. Lab values were significant for an elevated lactic acid, creatine kinase, and white blood cell (WBC) count (Table 1).

**Table 1:** Blood test results obtained from the patient following rapid response team activation.

Lab test	Lab value	Reference range
Sodium	140	136 - 145mmol/L
Potassium	3.8	3.6 - 5.3mmol/L
Chloride	103	98 - 110mmol/L
Bicarbonate	23.5	20.0 - 31.0 mmol/L
Blood urea nitrogen	25.0	6.0 - 24.0 mg/dL
Creatinine	1.26	0.5 - 1.0mg/Dl
Aspartate transferase (AST)	60	10 - 36 U/L
Alanine transferase (ALT)	29	6 - 29 U/L
Lactic acid	3.65	0 - 2 mmol/L
Creatine Kinase	1353	52 - 336 U/L
White blood cells	17.8	3.5 - 10.50K/µL
Hemoglobin	14.2	13.5 - 17.5 g/dL

A non-contrast CT of the head and cervical spine and maxillofacial CT returned negative for any acute process. The patient was given 25 mg of diphenhydramine and 1 mg of benztropine intramuscularly due to lack of intravenous access at the time. The patient’s symptoms did not resolve. An hour later, he was given an additional 25 mg of intravenous diphenhydramine with complete resolution of symptoms. An electroencephalogram was performed and did not demonstrate any epileptiform activity.

Following the episode, the patient’s oral aripiprazole was held. The patient was transferred to the medical floor for treatment of rhabdomyolysis with intravenous fluids. After aripiprazole was held, the patient did not experience any further extrapyramidal symptoms, including OGC or any other acute dystonic reaction. The patient's WBC count normalized the day after the rapid response without the use of antibiotics. After treatment for the rhabdomyolysis, the patient was returned to the inpatient psychiatric unit. He was placed on an alternate regimen for his labile mood and suicidal ideation, switching aripiprazole for quetiapine. With the new regimen, the patient’s mood stabilized. Following mood stabilization, the patient was safely discharged back home without any known recurrence of OGC six months following the incident.

## Discussion

OGC is a potential side effect primarily seen with anti-epileptics, selective serotonin reuptake inhibitors, tricyclic antidepressants, and high-potency neuroleptic agents [9]. It is rarely reported with aripiprazole. Certain factors increase the risk of OGC, such as young age, increased disease burden, personal or family history of acute dystonia, recent cocaine abuse, sudden discontinuation of anticholinergic medications, and certain metabolic disorders such as hyperthyroidism and hyperparathyroidism [10-12].

The diagnosis of OGC is clinical. In the setting of antipsychotic usage, medication-induced dystonia should be considered after excluding other potential underlying causes. With regards to our patient, he developed OGC after the aripiprazole dose was increased despite being on benztropine. We suspect the dosage increase likely triggered the acute dystonic reaction. Similarly, Ruiz de Villa et al. describe a case report of a patient who had OGC due to risperidone while on benztropine [10]. Thus, it was hypothesized at that time that benztropine prevention of extrapyramidal symptoms may be dose-dependent. Our patient may have needed a higher dose of benztropine, especially after the aripiprazole dosage was increased. Further studies are needed to assess the required benztropine dosage to prevent antipsychotic-induced extrapyramidal symptoms.

Specifically pertaining to our patient, his greatly elevated creatine kinase (CK) level of 1353 U/L and his lactic acid of 3.65 mmol/L may indicate that he suffered additional dystonic reactions prior to the arrival of medical personnel. It would be unusual for isolated OGC to elevate CK and lactic acid levels to this extent. Alternatively, his rhabdomyolysis may have been caused by the fall itself.

In general, treatment of OGC includes removing the offending agent. If discontinuing the medication is difficult, dose reduction should be sought. Furthermore, benztropine and diphenhydramine or other first-generation antihistamines may be given acutely to abort the symptoms. Benztropine may even be given prophylactically in patients on antipsychotic medications as seen with our patient to prevent the onset of OGC or other extrapyramidal symptoms.

## Conclusions

In conclusion, clinicians should be vigilant of OGC when giving aripiprazole. With this case report, we aim to increase awareness of aripiprazole-induced OGC. After ruling out other potential causes, OGC can be diagnosed and should be treated promptly with first-generation antihistamines and benztropine to avoid further complications and discomfort for the patient.
